# Feasibility of the TAMIS technique for redo pelvic surgery

**DOI:** 10.1007/s00464-016-4889-7

**Published:** 2016-04-11

**Authors:** W. A. A. Borstlap, N. Harran, P. J. Tanis, W. A. Bemelman

**Affiliations:** 1 Department of Surgery, Academic Medical Center, University of Amsterdam, PO Box 22660, 1100 DD Amsterdam, The Netherlands; 2 Department of Surgery, Donald Gordon Medical Centre, Johannesburg, South Africa

**Keywords:** Redo surgery, TAMIS, Rectal surgery

## Abstract

**Aim:**

The aim of this study was to report on the feasibility of transanal minimally invasive surgery (TAMIS) as a novel approach to redo colorectal or ileoanal anastomoses.

**Methods:**

From October 2014, a prospective institutional database was created for all consecutive patients who underwent redo surgery by TAMIS for presacral sinus or anastomotic stenosis after low anterior resection or pouch-related problems following restorative proctocolectomy. Intra-operative feasibility, 30-day postoperative outcomes, intestinal continuity and complications after 6-month follow-up were evaluated.

**Results:**

Of 17 included patients, 14 underwent anastomotic reconstruction and three completion proctectomy. The median operation time was 265 min (range 201–413). A successful rendezvous with simultaneous transabdominal access was achieved in 15 patients, and the procedure was completed by TAMIS alone in two. Five patients were readmitted within 30 days (29 %). Two (14 %) patients developed an anastomotic leakage within 30 days and 4 (24 %) developed a pelvic abscess requiring reintervention. One patient developed an urethra stenosis and was managed with a suprapubic catheter. Median follow-up was 9 (6–15) months. Within 6-month follow-up, the redo-TAMIS 1 patient developed a delayed anastomotic leak and 1 patient had a recurrent presacral abscess after stoma closure. Intestinal continuity was reached in 71 % of the patients at 6-month follow-up.

**Conclusion:**

TAMIS is a valuable approach in redo pelvic surgery, but is still associated with high complication rates related to the complexity of the underlying problem.

## What does this paper add to the literature?

This study describes the first consecutive cohort of patients undergoing redo pelvic surgery using transanal minimally invasive surgery (TAMIS) for the reconstruction of a low colonic anastomosis or an ileoanal pouch.

Transanal minimally invasive surgery (TAMIS) is slowly being incorporated into the colorectal surgeon’s armamentarium as an approach to pelvic dissection. Since its first description as an alternative to transanal endoscopic microsurgery (TEM) for local excision of rectal tumours, the advantages and its place in surgery for benign and malignant indications have been debated and discussed [1]. The use of conventional laparoscopic instruments and experience gained with transabdominal single port surgery has facilitated further development of the TAMIS technique, expanding its indications since 2009. The spectrum of pathology that can be managed with TAMIS has broadened from excision of intraluminal small rectal lesions to a full total mesorectal excision (TME) [2, 3].

One of the technical problems in redo pelvic surgery is to achieve adequate exposure. In addition, redo anastomotic surgery for patients with a chronic presacral sinus after low anterior resection or pouch dysfunction is often characterised by difficult dissection because of adhesions (inflammatory), fibrosis and distortion of the anatomical planes. We propose that the “bottom-up” minimally invasive approach provides increased accessibility and improved visibility compared to conventional approaches for the most distal part of the dissection in the pelvis.

The aim of this study was to report on the feasibility of the TAMIS approach for redo pelvic surgery based on the possibility to achieve a rendezvous with simultaneous transabdominal access at a predefined level. Next to 30-day postoperative outcomes, continuity and complications at 6 months after the redo-TAMIS will be analysed.

## Methods

From October 2014, all consecutive patients who underwent redo pelvic surgery via a minimally invasive transanal approach with a single port (GelPOINT^®^ Path Transanal Access Platform, Applied Medical, Rancho Santa Margarita, United States) were included. All procedures were performed by one of two consultants at the Academic Medical Centre, Amsterdam, with extensive experience in minimally invasive and redo colorectal surgery. The patient and treatment characteristics were retrospectively collected from the patient records. Operative, pathology, endoscopic and radiology reports as well as the patients charts were searched for patient demographics, primary treatment characteristics, tumour characteristics, hospital stay, preceding interventions or reinterventions (radiological, endoscopic and surgical) and disease status at the last date of follow-up.

### Surgical technique

**Fig. 1:**
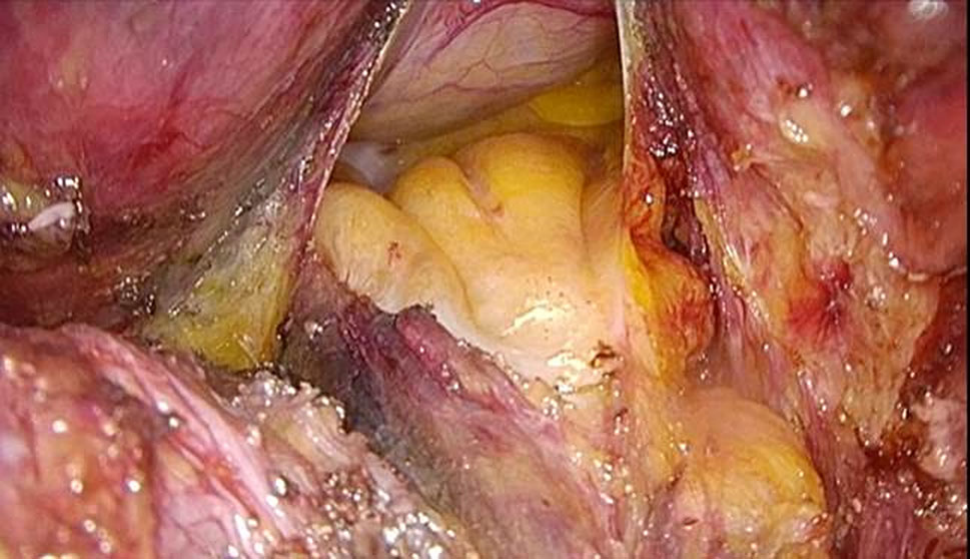
Rendezvous: Bottom-up

**Fig. 2:**
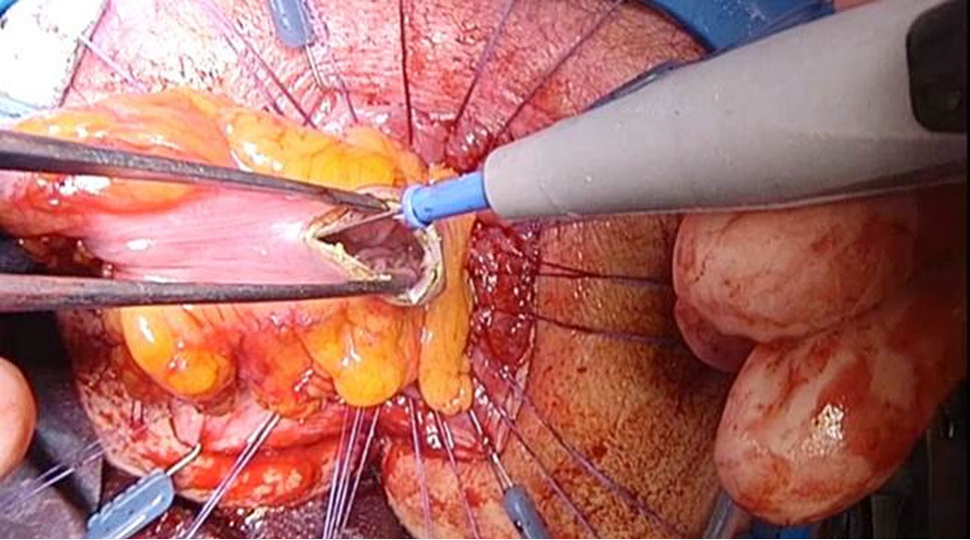
Pulled-through neorectum

For technical details of the TAMIS approach, we refer to the description by Attalah in 2009 [1]. The pneumoperitoneum created by the single port (GelPOINT^®^ Path Transanal Access Platform) enables increased accessibility to commence the dissection around the dehisced anastomosis. A purse-string suture of the neorectum was not always needed or possible when the leaking anastomosis was very low. In pelvic sepsis, the area is contaminated, so a purse string cannot prevent contamination of the wound bed anymore. Only if there is still a considerable amount of rectum (2–3 cm above the dentate line), then a purse string is feasible. The rectum is transected prior to potential placement of the purse-string suture in the proximal bowel. Feasibility of the TAMIS approach in this study was defined as the ability to complete a rendezvous from the “bottom-up” or transanal approach towards the “top-down” or abdominal approach at the level of the seminal vesicles in men or at the level where the neorectum curves anteriorly in women, beyond the leaking anastomosis (Fig. 1). By completing the rendezvous from the pelvis to the abdominal cavity in this manner, a canal is created where, after mobilisation of the splenic flexure, the colon or the reconstructed pouch can be exteriorised (Fig. 2). When the TAMIS-redo was performed completely transanal, there was no abdominal mobilisation. By completing the majority of the dissection from below, the extent of the proximal dissection is limited. Redo-TAMIS was used with the intention to limit “blind dissection” from the top and to achieve a safer and better visualised dissection of the dehisced anastomosis with an associated lower risk of nerve injury and bleeding.

After completion of the rendezvous, the single port is removed and the neorectum can be exteriorised for a tensionless hand-sewn coloanal anastomosis, using the Lone Star Retractor (Cooper Surgical, Trumbull, United States), and the hand-sewn anastomosis technique is previously described by Lacy and Penna et al. [3–5]. (Fig. 2). Anastomotic leakage was defined, as proposed by Caulfield, as a disruption of the anastomosis identified at reoperation or extravasation of contrast medium at the anastomotic site on an imaging study, irrespective of the presence of symptoms [6]. Subsequently, an abdominal abscess or free pelvic fluid collection without extravasation of contrast medium was considered an occult anastomotic leak. A chronic presacral sinus was defined as a sinus that persisted for more than 1 year from prior operation and had been confirmed by radiological imaging or endoscopically. All anastomosis are controlled with a sigmoidoscopy 2 weeks after the redo surgery. This in order to facilitate early salvage of the anastomosis with the endosponge. However, if there were clinical and/or haematologic signs of a leakage/infection, a CT-abdomen with rectal contrast is performed. We divided the postoperative course in “operative outcomes” (within 30 days from TAMIS-redo) and “postoperative outcomes” (complications that occurred between 30 days and 6 months from TAMIS-redo). Continuity was assessed after a follow-up period of 6 months

### Statistical analysis

For non-normal distributed data, medians with range are reported. All analyses were performed with IBM SPSS Statistics, version 20.0.0 (IBM Corp., Armonk, NY, United States).

## Results

### Patient characteristics

From October 2014 to June 2015, 17 patients underwent redo surgery via TAMIS: a coloanal or an ileo-pouch anal anastomosis (IPAA) in 14 patients and a completion proctectomy with end colostomy in three patients. Median age was 56 years (range 30–67). The underlying pathology was rectal carcinoma (*n* = 10, 59 %), ulcerative colitis (*n* = 6, 35 %) or familial adenomatous polyposis coli (FAP) (*n*=1, 7 %). The median number of procedures prior to redo surgery was 4 (range 1–22) (Table 1). Patient characteristics are shown in Table 2. All patients with rectal carcinoma had received neo-adjuvant treatment. The primary procedure performed was a low anterior resection (LAR) in ten patients and proctocolectomy with IPAA in seven patients. Thirteen (76 %) patients had a diverting stoma at the time of surgery. The diverting stoma was formed during the TAMIS procedure in 5 of 13 (38 %) patients, at the initial procedure in 3 (23 %) and at a reintervention prior to the TAMIS in 5 (38 %) patients. The four patients without a diverting ileostomy had pouch dysfunction instead of a history of anastomotic leakage, due to reasons described in Table 1. Simultaneous transabdominal access during TAMIS was gained via a laparotomy (*n* = 9), Pfannenstiel incision (*n* = 3) or laparoscopic (*n* = 3) depending on the approach of the initial operation. In two patients, the procedure was performed completely transanal.

**Table 1:** Indications for redo-TAMIS procedure

	Underlying disease	Indication for TAMIS	Number of interventions prior to TAMIS^a^	Type of interventions prior to TAMIS
Anastomotic problems: 13/17(76 %)				
1	*Carcinoma*	Presacral abscess	4	LAR (laparoscopic), endosponge, Transanal closure of defect, endosponge
2	*Carcinoma*	Stenosis of anastomosis	1	LAR (laparoscopic)
3	*Carcinoma*	Presacral sinus	4	LAR (open), relaparotomy, ileostomy closure, endosponge
4	*Carcinoma*	Presacral sinus	3	LAR (open), Ileostomy closure, relaparotomy with formation of new anastomosis
5	*Carcinoma*	Presacral sinus	5	LAR(laparoscopic), transanastomotic drain, ileostomy closure, endosponge, transanal closure of anastomotic defect.
6	*Carcinoma*	Presacral sinus	3	LAR (laparoscopic), endosponge, transanal closure of defect
7	*Carcinoma*	Presacral sinus	1	LAR (open)
8	*Carcinoma*	Presacral sinus	2	LAR (open), percutaneous abscess drainage.
9	*Carcinoma*	Presacral sinus presenting as a rectovaginal fistula	5	LAR (open), ileostomy closure, ileostomy formation due to leaking blind loop, JJ-Catheter placement, ileostomy closure
10	*Carcinoma*	Presacral sinus with enterocutaneous and small bowel fistula and an anastomotic stenosis	8	LAR (open), Ileostomy closure, trocar herniation correction, relaparotomy due to ileus, resection leaking anastomosis and end colostomy, parastomal hernia correction, surgically placed abdominal drain, endosponge
11	*Ulcerative colitis*	Presacral sinus presenting as a pouch fistula	22	Subtotal colectomy (open), relaparotomy+ ileostomy formation, ileostomy closure, proctectomy, relaparotomy, angiogram with coiling (2×), abdominal mesh placement, relaparotomy, Percutaneous drainage, endosponge, revision abdominal mesh + VAC abdomen, endosponge, transanal pouch revision, endosponge, transanal pouch revision, ramirez-plasty + pouch excision and formation new pouch, transanal closure anastomotic leakage, endosponge, transanal closure anastomotic leakage, endosponge, transanal closure anastomotic leakage
12	*Ulcerative colitis*	Presacral sinus presenting as a perianal fistula and cuffitis	3	Subtotal colectomy with J-pouch (laparoscopic), proctectomy, ileostomy closure, mesh removal + closure abdominal wall, relaparotomy + VAC abdomen, transanal pouch revision (2×),
13	*Ulcerative colitis*	Dehiscence of the posterior part of the IPAA	4	Subtotal colectomy without anastomosis (open), secondary IPAA, shortening of blind loop and formation of new IPAA, ileostomy formation
Pouch problems: 4/17 (23 %)				
14	*Ulcerative colitis*	Efferent loop syndrome	6	Subtotal colectomy with J-pouch (open), Correction of abdominal scar, endoscopic dilation of anastomosis (4×)
15	*FAP*	Obstructive polyp on pouch	6	Subtotal colectomy with J-pouch (open), partial jejunal resection, proctectomy, relaparotomy with formation ileostomy, ileostomy closure, transanal pouch revision
16	*Ulcerative colitis*	Voiding disorder pouch	2	Subtotal colectomy without anastomosis (open), secondary IPAA,
17	*Ulcerative colitis*	Ulcer on pouch leading to recurrent cuffitis	3	Proctocolectomy due to pancolitis (laparoscopic), ileostomy closure

^a^Including the primary procedure

**Table 2:** Patients characteristics

	*N* = 17
*Gender*	
Males (*n*, %)	10 (58)
*Age*	
Median age (years, range)	56 (30–67)
*ASA*	
I (*n*, %)	3 (18)
II (*n*, %)	13 (76)
III (*n*, %)	1 (6)
*BMI*	
Median BMI (range)	23.4 (18.6–33.6)
*Neo-adjuvant treatment*	
Any neo-adjuvant treatment (*n*, %)	10 (59)
Short course 5 × 5 Gy (*n*, %)	4 (24)
Long course with concomitant chemotherapy (*n*, %)	6 (35)
*Primary surgery*	
Low Anterior Resection with diverting ileostomy (*n*, %)	10 (59)
Proctocolectomy with IPAA (*n*, %)	7 (42)
*Approach of primary surgery*	
Open	11 (65)
Laparoscopic	6 (35)
*Time interval*	
Median Time Between Initial Procedure-TAMIS (Months, range)	49 (11–372)
*Earlier interventions*	
Median number of interventions prior to TAMIS	3 (1–21)
Of which Surgical (median, range)	61/82 (74 %)
Of which Radiological (median, range)	5/82 (6 %)
Of which Endoscopic (median, range)	16/82 (20 %)

#### Indications for redo anastomotic surgery

The indications for the redo surgery are listed in Table 1. They are divided into two groups: anastomotic problems (13/17) and pouch problems (4/17). In the anastomotic problems group, ten patients had a chronic presacral sinus due to prior anastomotic dehiscence after LAR, one patient had a presacral abscess, one a dehiscence of the posterior part of the IPAA, and the last patient had an anastomotic stenosis. In three out of the nine patients with a presacral sinus, the sinus presented as a perianal fistula originating from the previous anastomotic site. Problems of the pouch were caused by efferent loop syndrome, recurrent polyps from FAP, recurrent cuffitis of the pouch and pouch dilatation with a voiding disorder.

### Surgical outcomes

A successful rendezvous was achieved in 15 patients. In two patients, abdominal access was not required; adequate mobilisation (sleeve advancement) of the neorectum was achieved from the bottom-up approach alone. No operative deaths occurred. Median surgical time was 265 min (220–413). One intra-operative complication occurred (6 %): an injury of the right hypogastric vein, which was adequately controlled at the time of surgery. This patient did not require a blood transfusion and was discharged home after 4 days.

In all 14 patients, a hand-sewn anastomosis was constructed, a coloanal in seven and ileoanal in the other seven. In three patients, the anastomosis was not reconstructed.

The indications for the three patients with an end colostomy, and no continuity, were as follows. One patient had a large cavity identified prior to redo surgery and as such a predicted high likelihood of anastomotic failure; therefore, an omentoplasty was used to fill the remaining cavity and an end colostomy was constructed. The other patient without continuity had undergone six surgical abdominal reinterventions prior to presentation, including a Hartmann procedure due to a presacral sinus after the earlier LAR. He presented with a fistula from the presacral sinus to the rectal stump. As such it was decided, not to restore continuity. The third patient without continuity had chronic presacral sinus that presented as an enterocutaneous fistula and concomitant small bowel fistula; therefore, it was decided to resect the anastomosis, fill the remaining cavity with an omentoplasty, and to construct an end colostomy.

#### Operative outcomes

One patient developed a urethra stenosis, which was successfully managed with a cystoscopic intervention by the urologist 2 months after the redo-TAMIS. Median postoperative hospital stay was 8 days (range 4–23). Two out of 14 (14 %) patients developed an anastomotic leak within 30 days. The first case was managed transanally with reinforcement of the anastomotic defect and debridement of the area of the leak. The second patient required a revision of the reconstructed IPAA 17 days following the primary procedure, for which the TAMIS approach was used as well.

**Fig. 3:**
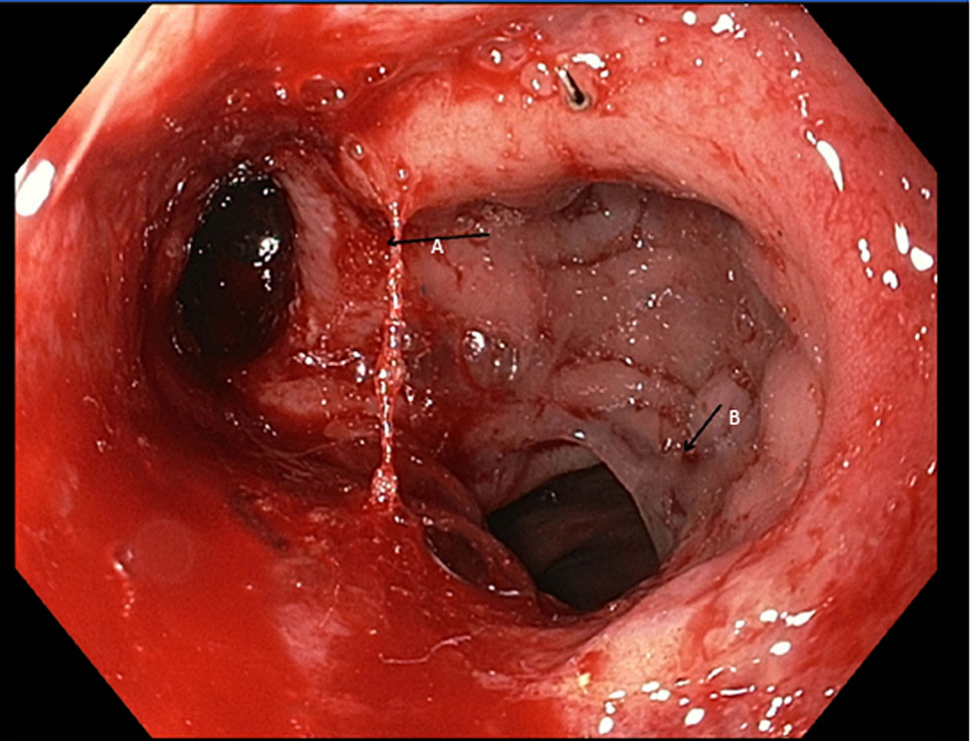
Dehiscent anastomosis**. A** anastomotic defect. **B** descending colon

Using CT and sigmoidoscopy, 4 patients (24 %) were diagnosed as having a pelvic abscess that initially was not in continuity with the anastomosis. All patients with a pelvic abscess were symptomatic (leucocytosis/fever/ileus). This occurred 7, 12, 14 and 21 days after the TAMIS. These were scored as occult anastomotic leakages, as proposed by Caulfield [6]. In three patients, the abscess was drained percutaneously. In the remaining patient, the abscess was localised in the pouch of Douglas which was insufficiently drained via a percutaneous abdominal approach. Subsequently, the abscess eroded through the anastomotic site 5 days after placement of the abdominal drain. A picture of this defect is enclosed in Fig. 3. This patient was managed via Endosponge (B-Braun Medical B.V., Melsungen, Germany), followed by transanal closure of the anastomotic defect. The endosponge was changed every 3 days until the cavity was free from debris. The patient required five endosponge changes until the abscess cavity was deemed suitable for primary closure. The defect was closed transanally using the Lonestar retractor (Cooper Surgical, Trumbull, United States).

#### Postoperative outcomes

The diverting ileostomy was successfully closed in 7 of 10 (70 %) of the patients at 6 months. Of these three remaining patients, one patient did not have the ileostomy closed due to logistic reasons. The second patient had presacral abscess within 30 days, which was managed with a percutaneous drain; however, at 6 months, it was deemed too early for stoma closure. The third patient developed a recurrent presacral abscess following ileostomy closure requiring a quaternary ileostomy and endosponge treatment of the abscess cavity. Other than a patient with a postoperative anaemia treated with a blood transfusion, no complications occurred following stoma closure. Of the four patients with pouch problems that underwent a redo-TAMIS without defunctioning ileostomy, one patient required an ileostomy 3 months after the redo-TAMIS due to faecal incontinence. Concluding, continuity was reached in 10 of 14 (71 %) of the patients following redo-TAMIS.

Other than the two cases with a complicated course described above, one patient developed a late anastomotic leakage, 32 days after the redo-TAMIS, which was managed by a redo-TAMIS and his ileostomy was reversed successfully within 6 months. No further complications occurred between 30 days and 6 months of follow-up.

The postoperative outcomes are summarised in Table 3.

**Table 3:** (Post)Operative outcomes

Length of in hospital stay (median, range)	8 (4–23)
Any Postoperative complications (within 30 days)	9 (53)
Clavien–Dindo III or higher	7 (41)
Anastomotic Leakage (*n*, %)	2 (14)
Occult leakage (Abdominal abscess)	4 (24)
Ileus (*n*, %)	2 (12)
Urethra stenosis	1 (6)
Dehydration	1 (6)
Readmissions (within 30 days)	5 (29)
*Cause readmission*:	
Anastomotic leakage:	2
Abdominal abscess	1
ileus	1
Dehydration	1
Continuity at 6 months post redo-TAMIS	10 (71 %)
*Postoperative complications from 30 days to 6 months*	3 (18 %)
Faecal incontinence requiring diverting ileostomy	
Recurrent presacral abscess following stoma closure requiring new ileostomy and prolonged endosponge treatment	
Delayed anastomotic leakage (32 days post-TAMIS)	
*Complications following stoma closure*	2 (22 %)
Recurrent presacral abscess	
Postoperative anaemia requiring blood transfusion	
*Length of follow-up (median, months)*	9 (6–15)

## Discussion

**Fig. 4:**
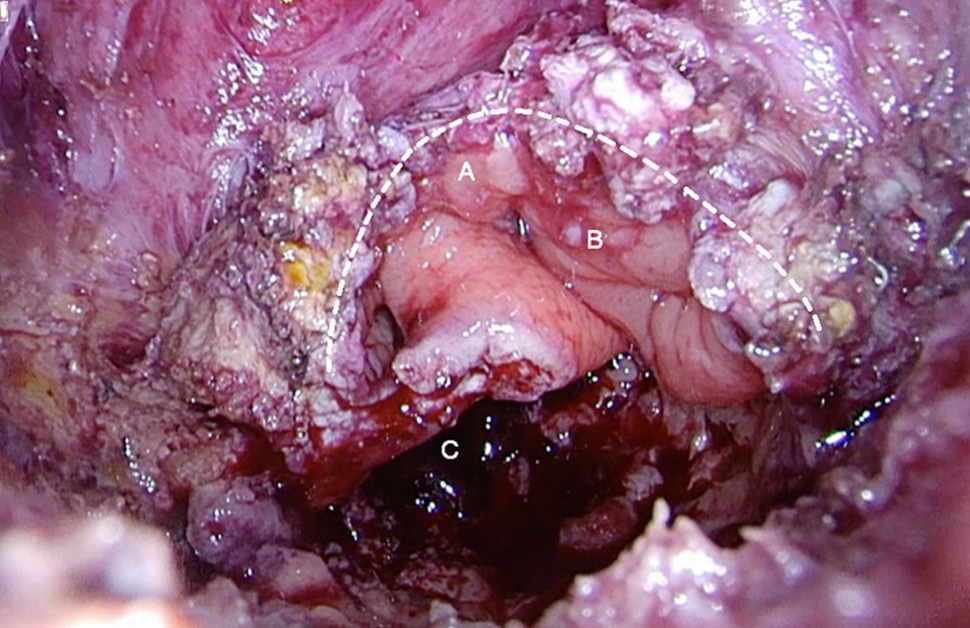
Abscess cavity with well-vascularised colon beyond the anastomotic defect. *Dotted line*: Dissection plane, **A** Blind ending loop, **B** Descending colon, **C** Abscess cavity

The present study indicates that the TAMIS technique is feasible and useful for redo pelvic surgery following LAR or IPAA. Performing the otherwise troublesome low pelvic dissection via the bottom-up approach facilitates the exposure of an area that is difficult to access transabdominally due to altered anatomy, chronic inflammation related to anastomotic insufficiency, prior surgery with scar tissue and preoperative radiotherapy (Fig. 4). In all patients, it was possible to reach the dissection point, where the neorectum curves anteriorly, enabling a rendezvous with the top-down abdominal approach. This facilitated completion of the dissection of the neorectum from the top-down.

In current literature, the feasibility of the TAMIS technique for TME is being presented [2, 7–12]. The future sustainability of the TAMIS technique will be determined by the quality of the surgery which can be assessed by TME specimen grading, the circumferential margin and the long-term oncologic outcomes. These results are still awaited. In redo surgery for low coloanal or ileoanal anastomoses, there are no oncological issues. As such, the TAMIS technique, for this indication, should therefore be evaluated based on the feasibility of the procedure and perioperative complication rates [13]. Applying the TAMIS approach allowed for a successful rendezvous in all patients in whom a simultaneous transabdominal access was required and TAMIS alone was appropriate in the remaining two patients. Using conventional open surgery for redo pelvic procedures, the surgeon performs the deep pelvic dissection and anastomosis with limited vision and tactile guidance. The pneumatic insufflation in combination with a magnified endoscopic image and the possibility of 30° angulation, afforded by the TAMIS technique, improves visualisation and surgical access and as such has the potential to revolutionise the approach to redo low anastomotic surgery and extensive sleeve advancement of the pouch. Furthermore, the advantage of TAMIS-redo surgery is that the neorectum is used to guide the dissection, thereby potentially minimising the chance of nerve and vascular injury deep in the pelvis. Potential nerve injury has to be evaluated in further studies using validated scoring systems and questionnaires to assess pre- and postoperative bladder, sexual and bowel dysfunction in patients undergoing redo pelvic surgery. Pelvic abscesses seem to be common either due to anastomotic insufficiency or due to recurrence of the abscess at the former site of the presacral sinus. Endosponge-assisted drainage and salvage of the anastomosis seems useful [14]. It is important to highlight a common problem experienced during many of the TAMIS procedures related to the access channel (GelPOINT^®^ Path Transanal Access Platform). The proximal lip of the platform needs to be secured above the levator ani muscle to ensure it remains in situ and creates an adequate air seal. In males, this positioning was particularly challenging due to their anatomically long anal canal and the narrow space between the ischial bones. When this was not achieved well, it resulted in a poor air seal and subsequently excessive movement of the platform with decreased visibility and control. Additionally, there is a concern that stretch placed on the anal sphincter by the platform could impact continence, especially when a coloanal anastomosis was performed. For these reasons, we apply as a routine a pudendal nerve block to ensure optimal relaxation of the external anal sphincter. A feared complication of the TAMIS technique is injury of the urethra as the urethra occasionally is difficult to identify with the transanal approach. One patient in our cohort experienced a urethral stenosis. It was postulated that this stenosis was caused by the heat produced by the diathermy. Another complication that should be cautioned is bleeding and nerve injury due to dissection outside the endopelvine fascia. The literature on redo low anastomotic surgery is limited to small retrospective cohort series. These series only report the outcomes of open surgery. Of interest when comparing a similar cohort of patients, the median time of surgery in our study was 265 min, substantially shorter than the 435 min reported by Genser et al. [15]. They reported a continuity rate of 88 %, which is higher than our reported 71 %. However, we analysed continuity after 6 months and Genser et al. after a median of 21 months, so therefore a lower rate can be explained by this shorter follow-up period. In contrast to the use of TAMIS for TME surgery, there are still no reports in the literature documenting its application for redo surgery. Because it is our feeling that TAMIS can be of additional value for this indication, we wanted to share our experience. We acknowledge that the technique is new with a high level of complexity. This procedure should only be performed in centres with extensive experience in minimally invasive as well as redo colorectal surgery and should be monitored by adequate prospective registration of intra-operative findings and postoperative outcome. This may result in better defining the role of TAMIS for anastomotic reconstruction and other complex pelvic procedures.

## Abbreviations

APR: Abdomino perineal resection
CD: Crohns disease
FAP: Familial adenomatous polyposis coli
IAPA: Ileoanal Pouch anastomosis
IBD: Inflammatory Bowel Disease
LOS: Length of Stay
LAR: Low anterior resection
SILS: Single incision laparoscopic surgery
TAMIS: Trans anal minimally invasive surgery
TME: Total mesorectal excision
UC: Ulcerative colitis
CT: Computed tomography
IQR: Interquartile range
